# Removal Efficiency of Cr^6+^ by Indigenous *Pichia* sp. Isolated from Textile Factory Effluent

**DOI:** 10.1100/2012/708213

**Published:** 2012-05-02

**Authors:** Pablo M. Fernández, María M. Martorell, Julia I. Fariña, Lucia I. C. Figueroa

**Affiliations:** ^1^Planta Piloto de Procesos Industriales Microbiológicos PROIMI-CONICET, Avenida Belgrano y Caseros, Tucumán T4001MVB, Argentina; ^2^Microbiología Superior, Facultad de Bioquímica, Química y Farmacia, Universidad Nacional de Tucumán UNT, Tucumán 4000, Argentina

## Abstract

Resistance of the indigenous strains *P. jadinii* M9 and *P. anomala* M10, to high Cr^6+^ concentrations and their ability to reduce chromium in culture medium was studied. The isolates were able to tolerate chromium concentrations up to 104 **μ**g mL^−1^. Growth and reduction of Cr^6+^ were dependent on incubation temperature, agitation, Cr^6+^ concentration, and pH. Thus, in both studied strains the chromium removal was increased at 30°C with agitation. The optimum pH was different, with values of pH 3.0 and pH 7.0 in the case of *P. anomala* M10 and pH 7.0 using *P. jadinii* M9. Chromate reduction occurred both in intact cells (grown in culture medium) as well as in cell-free extracts. Chromate reductase activity could be related to cytosolic or membrane-associated proteins. The presence of a chromate reductase activity points out a possible role of an enzyme in Cr^6+^ reduction.

## 1. Introduction

Heavy metals found in wastewaters are harmful to the environment and their effects on biological systems are very severe. Chromium is one of the most widely used metals in industry, such as steel production, alloy preparation, wood preservation, leather tanning, metal corrosion inhibition, paints pigments, metal plating, tanning, and other industrial applications [1]. Chromium exists in several oxidation states from Cr^2+^ to Cr^6+^. In nature, trivalent and hexavalent forms are the dominant oxidation states. The toxicity of chromium is dependent on its oxidation state, Cr^3+^ is rather benign and easily adsorbed in soils and waters; whereas Cr^6+^, which is the toxic form, is not readily adsorbed and is soluble [2]. Thus, Cr^6+^, a carcinogenic element, is highly toxic to all forms of life but Cr^3+^, an essential micronutrient for many higher organisms, is relatively insoluble in water and 100 times less toxic than Cr^6+^ [3]. Chromium hexavalent toxicity is believed to be caused by the negatively charged chromate oxyanion, which can be easily transported into microbial cells. Once inside the cells, the oxyanion is believed to undergo immediate reduction reactions leading to the formation of various reactive intermediates, which are harmful to the cell organelles, proteins, and nucleic acids [4].

For that reason, it is important to develop an innovative, low cost, and ecofriendly method for the toxic heavy metal removal from the wastewater, instead of the conventional physical-chemical ones [1, 5]. Several microorganisms have the exceptional ability to adapt to and colonize the noxious metal-polluted environments. These microorganisms have developed the capabilities to protect themselves from heavy metal toxicity by various mechanisms such as adsorption, uptake, methylation, oxidation, and reduction.

Yeasts are known for playing an important role in the removal of toxic heavy metals [4, 6, 7]. Furthermore, the occurrence of indigenous Cr^6+^ reducing eukaryotic microorganisms, including those not related with Cr^6+^ contamination, has emerged as an important nonconventional yeasts-based bioremediation method with significant biological relevance and biotechnological applications.

Microbial Cr^6+^tolerance and Cr^6+^reduction are independent events. However, for the Cr^6+^-reduction cells must tolerate Cr^6+^, otherwise the cell growth is inhibited. Some authors argue that the microbial reduction of Cr^6+^ can be considered as an additional mechanism of resistance to chromate, which is usually not encoded in plasmids [8]. The enzymatic biospeciation of Cr^6+^ to Cr^3+^ with eukaryotic microorganisms was reported in *Candida maltose* [9], *C. utilis* [10], fungi *Hypocrea tawa* [11], and *Aspergillus* [12]. But it was not possible to continue with the purification and characterization of the protein involved, therefore available information is scarce. In this context, the study of specific chromate reductases is meaningful to understand the cellular mechanisms in future bioremediation processes.

The present study deals with the ability of *P. jadinii* M9 and *P. anomala* M10 to grow and remove chromium in batch cultures and using cell-free extracts. The effects of different factors on Cr^6+^ removal, including pH, temperature, agitation, and initial Cr^6+^ concentration were also considered and optimum removal parameters were established.

## 2. Materials and Methods

### 2.1. Yeast Strains and Culture Conditions

Chromate-resistant yeasts *Pichia jadinii* M9 and *Pichia anomala* M10, previously isolated from textile factory effluents (Tucumán, Argentina) were used [13]. For the inocula, the yeast strains were grown in 500 mL-Erlenmeyer flasks containing 100 mL of Czapek malta medium using methodology described by Fernández et al. [13].

Chromium removal experiments were performed using YNB' medium amended with Cr^6+^ and inoculated with a constant biomass. YNB' medium was chosen based on previous assays that confirmed lower interferences of this medium during Cr-bioremediation and Cr^6+^-quantification by 1,5-diphenylcarbazide (DPC) [14]. YNB' composition (in g L^−1^) was 10 × yeast nitrogen base (YNB w/o amino acids and ammonium sulfate; Difco), 10% (v v^−1^); sucrose, 50; ammonium sulfate, 0.6; pH 5.0. All the experimental sets were performed on a rotary shaker (250 rev min^−1^) at 25°C in 250 mL Erlenmeyer flasks containing 50 mL of culture medium, unless otherwise stated. 

The Cr^6+^ (as K_2_Cr_2_O_7_ or K_2_CrO_4_) stock solution (5,200 *μ*g mL^−1^) was prepared in bidistilled water and filter-sterilized (0.2 *μ*m-cellulose acetate membrane filter; Sartorius).

### 2.2. Effect of Cr^6+^ on Yeasts Growth

Chromate resistance test and growth curves were determined in YNB' medium supplemented with the desired Cr^6+^ concentration and without chromium (control). Growth was monitored at specific time intervals by biomass dry weight (BDW). Samples from culture were spun down at 10,000× g for 10 min. The distilled water suspended pellet was filtered through a 0.45 *μ*m cellulose acetate membrane filter (Sartorius) and dried at 85°C until constant weight to determine BDW in g L^−1^ [13]. For determination of Cr^6+^ concentration, a miniaturized protocol was developed as follows: to 50 *μ*L of sample supernatant, 50 *μ*L of 0.2 N H_2_SO_4_ were added and the volume was made up to 2 mL with distilled water. After mixing with 40 *μ*L of 5 mg DPC mL^−1^ acetone, the mixture was allowed to stand for 10 min and spectrophotometric determinations were performed at 540 nm (Beckman DU640) against a reagent blank. Cr^6+^ concentrations were quantified by the use of an external K_2_Cr_2_O_7_ standard with a 7-point calibration curve [14].

### 2.3. Factors Affecting Cr^6+^ Removal

To characterize the Cr^6+^-reduction efficiency by strains M9 and M10, the effects of temperature (10, 20, 25, 30°C), initial pH (3.0, 5.0, 7.0, 9.0), agitation (0, 150, 250 rev min^−1^), and initial Cr^6+^ concentration (26–104 *μ*g mL^−1^) were investigated. Cr^6+^ reduction was studied in aerobic batch cultures. The following set of standard conditions was chosen as the starting point: 52 *μ*g mL^−1^ of initial Cr^6+^ concentration, pH 5.0, 25°C and 250 rev min^−1^. Samples were withdrawn at defined times and analyzed for disappearance of Cr^6+^ as described above. In order to monitor any abiotic Cr^6+^ reduction, cell-free control experiments were carried out for each assayed condition.

### 2.4. Preparation of Cell-Free Extract and Enzymatic Determinations

To prepare the crude cell-free extract, the yeast cultures were grown in 200 mL YNB' medium for 48 h at 25°C with 52 *μ*g mL^−1^ Cr^6+^ and without chromium (control). Cells were harvested by centrifugation at 10,000 ×g for 10 min. Pellets were washed twice with 50 mM phosphate-citrate buffer (pH 5.0) and suspended in the same buffer with protease inhibitor cocktail (SET1; Calbiochem) plus a volume of sterilized glass beads. Cells were disrupted by sonication for 5 min in cold environment conidtions (5 cycles: 59 seg on, 30 seg off; Sonics Vibra Cell VCX 130). The homogenate was centrifuged at 10,000 ×g for 10 min at 4°C to remove cell walls and unbroken cells. The supernatant filtered through a 0.2 *μ*m cellulose acetate membrane filter was used as a crude extract and called cell-free extract (CFE). Decrease of chromate concentration by CFE was assayed after 30 min at 30°C using 50 *μ*L of sample preparation in 0.25 mL reaction mixtures containing (to a final concentration): 50 mM phosphate-citrate buffer (pH 5.0), 26 *μ*g mL^−1^ K_2_CrO_4_, 1 mM NADH; these concentrations were saturating and noninhibitory under these conditions. The reaction was started by addition of chromate to the reaction mixture. Hexavalent chromium was spectrophotometrically quantified, as previously described. Protein was determined using Bicinchoninic Acid Kit (BCA, Sigma), with BSA as standard.

## 3. Results and Discussion

### 3.1. Effect of Initial Cr^6+^ Concentration on Cells Growth

**Figure 1:**
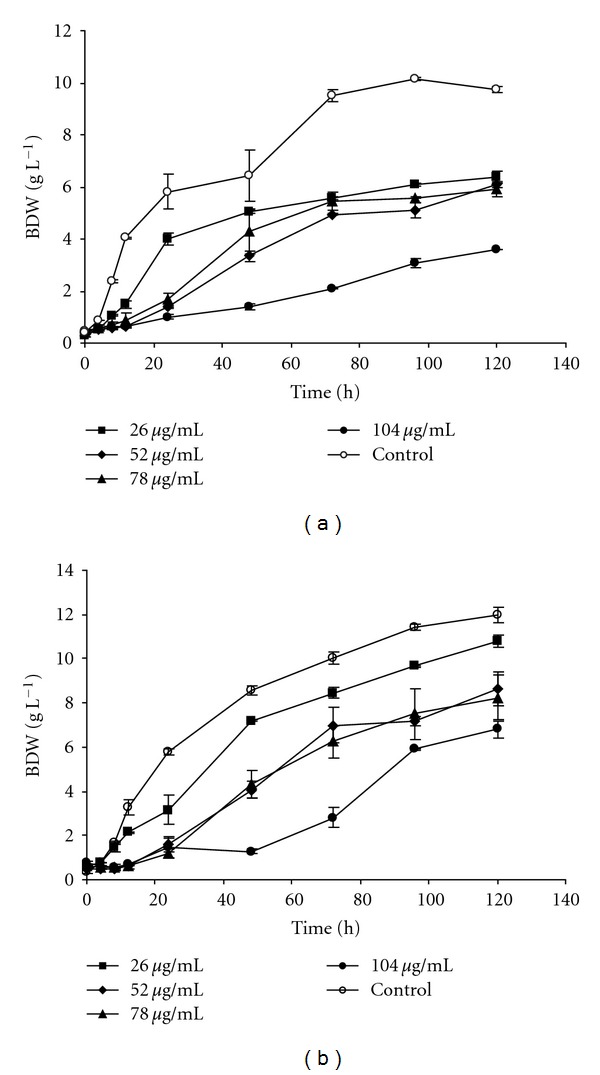
Growth curves of *P. jadinii* M9 (a) and *P. anomala* M10 (b) at varying Cr^6+^ concentrations as K_2_Cr_2_O_7._

Cr^6+^ resistance of *P. jadinii* M9 and *P. anomala* M10 was evaluated by growth response of the strains under different concentrations of Cr^6+^. Growth curves of yeast isolates with or without Cr^6+^ were plotted (Figures 1(a), 1(b)). The cells grew well in the medium with a range of initial Cr^6+^ concentration of 26–104 *μ*g mL^−1^. However, the growth curves of *P. jadinii* M9 and *P. anomala* M10 in the medium containing Cr^6+^ did not follow the same growth pattern as the control, indicating a possible toxic effect of Cr^6+^ on the cells. It was obvious that the growth of cells was heavily influenced by Cr^6+^ at a concentration of 104 *μ*g mL^−1^ (biomass concentration drop a 63% and 56% for *P. jadinii* M9 and *P. anomala* M10, resp.), but it did not suppressed the cells growth. The experiments conducted with Cr^6+^ concentrations of 26, 52, 78 *μ*g mL^−1^ had only slight effects on the growth (Figures 1(a), 1(b)). The *P. jadinii* M9 and *P. anomala* M10 strains completely reduced all Cr^6+^ concentrations tested; thus, overall efficiency of Cr^6+^ reduction (100%) was not affected by initial Cr^6+^ concentration. The highest concentration of Cr^6+^ (104 *μ*g mL^−1^) that allowed growth and was completely reduced by* P. jadinii* M9 and *P. anomala* M10 was much higher than concentrations commonly found to be reduced by bacteria [15], yeasts [9], and filamentous fungi [16]. However, it is important to consider that the microbial chromate-resistance and chromate-reduction parameters are correlated with medium composition and cell density [13]. The real toxicity of Cr^6+^ could be masked or underestimated due to complexation of Cr^6+^ with organic components. The minimal medium used in our study eliminated/minimized the possible complexation of Cr^6+^ with media components and allowed the assessment of the toxicity of Cr^6+^ more accurately.

In both strains, it was observed that, although residual Cr^6+^ concentration decreased as incubation progressed, total chromium in solution remained virtually constant (data not showed, Fernández et al., unpublished) and chromium did not accumulate in the cell, which indicates that *P. jadinii* M9 and *P. anomala* M10 were able to reduce chromium to forms of lower valency. Taking into consideration that the more stable forms of chromium are the trivalent and hexavalent ones [17], it seems most likely that the M9 and M10 strains were capable of transforming the highly toxic and soluble hexavalent chromium to the less toxic and mobile trivalent form.

**Figure 2:**
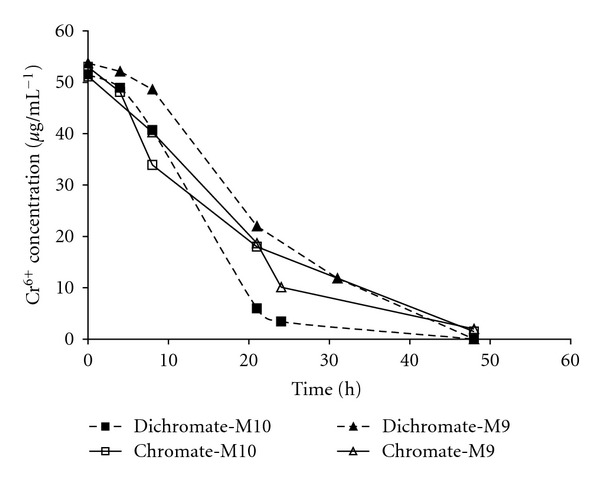
Cr^6+^-removal yield by *P. jadinii* M9 and *P. anomala* M10 exposed to different forms of Cr^6+^ (chromate: CrO_4_^−2^ and dichromate: Cr_2_O_7_^−2^) at 52 *μ*g mL^−1^ initial Cr^6+^ concentration during 48 h.

Hexavalent chromium reduction potential of *P. jadinii* M9 and *P. anomala* M10 was assessed with two kinds of Cr^6+^ salts, K_2_CrO_4_ (chromate), and K_2_Cr_2_O_7_ (dichromate). Cr^6+^ (at initial concentration of 52 *μ*g mL^−1^) was reduced up to 100% by both strains within 48 h (Figure 2). Importantly, Cr^6+^ occurs in aquatic environment either as CrO_4_^2−^ or Cr_2_O_7_^2−^ [18] and the strains used in this study were able to reduce both forms of hexavalent chromium.

### 3.2. Factors Affecting Cr^6+^ Reduction

The effect of initial Cr^6+^ concentration on Cr^6+^ reduction was investigated over a range of 26–104 *μ*g mL^−1^ under aerobic conditions. As shown in Table 1, Cr^6+^ reduction occurred even at the highest concentration of 104 *μ*g mL^−1^, and the time taken for total reduction of Cr^6+^ increased with increasing concentration of Cr^6+^. Complete Cr^6+^ reduction was observed at 96 and 72 h, for *P. jadinii* M9 and *P. anomala* M10, respectively. Megharaj et al. [19] also observed that the time required for total Cr^6+^ reduction increased with increasing initial Cr^6+^ concentration. The *Pseudomonad* strain CRB5 showed complete reduction of 20 *μ*g mL^−1^ of chromate after 120 h [18], whilst *B. sphaericus *AND303 failed to completely reduce 10 *μ*g mL^−1^ of Cr^6+^ [20].

Initial culture medium pH was considered as a relevant factor for growth and Cr^6+^ removal by strains M9 and M10. The time required for complete removal of Cr^6+^ in every experimental set is listed in Table 1. The optimum pH for the strain *P. jadinii* M9 was pH 7.0. In the case of *P. anomala* M10, the optimum pH for Cr^6+^ reduction was pH 3.0. Nonetheless, strain M10 was also capable of reducing Cr^6+^ in the range of 3.0–9.0 with an appreciable efficiency at neutral pH. Some authors have reported that reduction of chromium in various fungal strains, such as *Rhizopus nigricans* [21], *R. arrhizus* [22], and *Mucor hiemalis* [23] occurred at pH 2.0-3.0. It is known that a drop in pH causes the protonation of the adsorbent surface, inducing a strong attraction of negatively charged Cr^6+^-ions. Accordingly, biosorption increased with increasing acidity of the solution. The opposite would occur with increasing pH, inducing changes in the adsorbent surface, thereby preventing the Cr^6+^-ion biosorption. On the other side, Farrell and Ranallo [24] noted that in enzymatic Cr^6+^ reduction, changes in pH affect the degree of enzyme ionization, with protein conformation and enzyme activity modifications. This would explain why the acidity is not absolutely critical for a better Cr^6+^ removal. Related, *P. anomala* M10 showed two optimum pH values. The lowest (pH 3.0) could be related to stimulation of the biosorption phenomena, while pH 7.0 could be linked to improved enzymatic Cr^6+^ reduction. No measurable changes in Cr^6+^ concentrations were detected after 120 h of incubation in cell-free controls at the different pH values assayed. These results suggest that Cr^6+^ removal by medium components was not significant in these experiments and also indicate that Cr^6+^ reduction observed in the Cr^6+^ removal experiments conducted with cells was not due to the pH changes that occurred as result of metabolic activity of the growing cells.

Temperature was also an important factor on microbial Cr^6+^ removal. Chromate removal, by strains *P. jadinii* M9 and *P. anomala* M10 was evaluated under four different temperatures: 10, 20, 25, and 30°C for 120 h. These strains reduced Cr^6+^ in the culture medium more rapidly with an increment in temperature, with an optimum value of 30°C, as shown in Table 1. Generally, an increase in temperature increases the Cr^6+^-removal rate and reduces the contact time required for metal-removal, which is due to a direct increase in the rate of redox reaction [25]. Similarly, the optimum temperature for Cr^6+^ reduction by *Bacillus* sp. [26] and *Pseudomonad* strain CRB5 was 30°C [27].

The results of shaken versus stationary cultures are presented in Table 1. Generally, Cr^6+^ removal was enhanced by shaking the cultures, but strains *P. jadinii* M9 and *P. anomala* M10 could achieve a complete removal (100%) of the metal, both at stationary and shaken states. The aeration and the cell/metal contact are directly related to the removal of it. However, the alternative to remediate Cr^6+^ without agitation is particularly important for *in situ* bioremediation applications and may represent a valuable advantage from the economic point of view.

### 3.3. Chromate Reduction by Cell-Free Extract (CFE)

**Figure 3:**
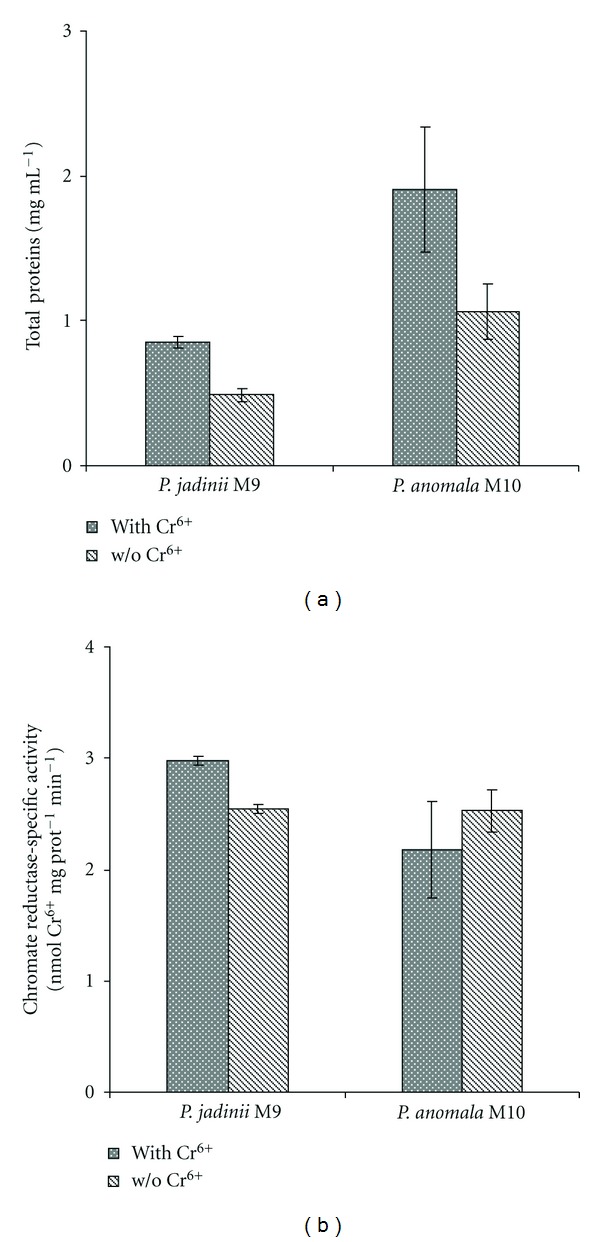
Total proteins (a) and chromate reductase-specific activity (b) in cell-free extract of *P. jadinii* M9 and *P. anomala* M10 grown with or without Cr^6+^. The reaction was started by addition of chromate, and the mixture was incubated at 30°C for 30 min.

Yeast cells recovered from cultures grown in the presence of 52 *μ*g mL^−1^ of Cr^6+^ and without Cr^6+^ (control) were tested for chromate reductase activity. The concentration of protein obtained in CFE from cultures with Cr^6+^ was two times higher than the control ones (Figure 3(a)). The chromate reductase specific activity in the CFE of *P. jadinii* M9 was higher in cultures with Cr^6+^, which could be interpreted as an induction by the metal present in the culture medium. In the case of *P. anomala* M10, there were no significant differences in chromate reductase specific activity between the different CFEs (Figure 3(b)). Das and Chandra [28] studied a strain of *Streptomyces* sp. M3 and noticed an increase in the chromate reductase activity when working in cultures with Cr^6+^. These same authors found that enzyme-expression was constitutive. Chromate reductase enzymes with constitutive expression were also discovered in *Bacillus* species [29, 30]. In the case of constitutive expression, it could be possible that the activity was not specific for this metal and, therefore, normally expressed in cells. It could also take place by induction of some other components of the culture medium with or without Cr^6+^. Kwak et al. [31] reported the presence of chromate reductase activity in *V. harveyi*, which also had nitroreductase activity. In *P. denitrificans*, the iron reductase (Ferb) also showed chromate reductase activity [32].

It is important to point out that the specific chromate reductase activity in the cells from cultures with Cr^6+^ could be masked by an increase in the concentration of other proteins not related with the metal reduction. That could be happening in the case of *P. anomala* M10 (Figure 3(b)). This protein could be part of a protective mechanism in response to the stress suffered in the presence of Cr^6+^. However, to date most of the proteins that undergo changes in presence of Cr^6+^ have not yet been identified, and therefore, its particular function could not be determined.

These data indicate that the chromate reductase activity present in CFE of *P. jadinii* M9 and *P. anomala* M10 could be related with cytosolic or associated membrane proteins, which in this respect resembles the activity found in chromate-resistant bacteria [30], and *Candida maltosa* RR1 [9].

## 4. Conclusions

Environmental isolates *P. jadinii* M9 and *P. anomala* M10 can be exploited for bioremediation of hexavalent chromium, since they are chromate-resistant yeasts and possess the capability to reduce the toxic hexavalent form to its nontoxic trivalent form. The results obtained may provide useful information for the removal of chromate under a wide range of environmental conditions. Systematic studies are needed to determine the real nature of activities so far called as chromate reductases. A future communication will deal with the chromate reductase activities characterization. This information will greatly facilitate the use of the involved proteins to enhance the chromate remediation potential of *P. jadinii* M9 and *P. anomala *M10.

## Figures and Tables

**Table 1:** The effect of factors on Cr^6+^ removal, including pH, temperature, agitation, and initial Cr^6+^ concentration.

Parameters	Time for complete Cr^6+^ removal (h)	
	Strains	
Initial Cr^6+^ concentration (*μ*g mL^−1^)	*P. jadinii* M9	*P. anomala* M10
26	12	8
52	48	24
78	48	48
104	96	72
Temperature (°C)		
10	+120	72
20	48	24
25	48	24
30	24	8
Agitation (rev min^−1^)		
0	48	72
150	48	24
250	48	24
pH		
3	+120	8
5	48	24
7	12	12
9	+120	48

Reference +120: complete Cr^6+^ removal was not achieved after 120 h culture.

## References

[1] Tseng JK, Bielefeldt AR (2002). Low-temperature chromium(VI) biotransformation in soil with varying electron acceptors. *Journal of Environmental Quality*.

[2] Kotaś J, Stasicka Z (2000). Chromium occurrence in the environment and methods of its speciation. *Environmental Pollution*.

[3] Morales-Barrera L, Cristiani-Urbina E (2006). Removal of hexavalent chromium by Trichoderma viride in an airlift bioreactor. *Enzyme and Microbial Technology*.

[4] Ksheminska H, Fedorovych D, Babyak L, Yanovych D, Kaszycki P, Koloczek H (2005). Chromium(III) and (VI) tolerance and bioaccumulation in yeast: a survey of cellular chromium content in selected strains of representative genera. *Process Biochemistry*.

[5] Juvera-Espinosa J, Morales-Barrera L, Cristiani-Urbina E (2006). Isolation and characterization of a yeast strain capable of removing Cr(VI). *Enzyme and Microbial Technology*.

[6] Guillén-Jiménez FM, Morales-Barrera L, Morales-Jiménez L, Hernández-Rodríguez CH, Cristiani-Urbina E (2008). Modulation of tolerance to Cr(VI) and Cr(VI) reduction by sulphate ion a *Candida* yeast strain isolated from tannery wastewater. *Journal of Industrial Microbiology and Biotechnology*.

[7] Villegas LB, Fernández PM, Amoroso MJ, De Figueroa LIC (2008). Chromate removal by yeasts isolated from sediments of a tanning factory and a mine site in Argentina. *BioMetals*.

[8] Cervantes C, Campos-García J, Devars S (2001). Interactions of chromium with microorganisms and plants. *FEMS Microbiology Reviews*.

[9] Ramírez-Ramírez R, Calvo-Méndez C, Ávila-Rodríguez M (2004). Cr(VI) reduction in a chromate-resistant strain of *Candida maltosa* isolated from the leather industry. *Antonie van Leeuwenhoek*.

[10] Muter O, Patmalnieks A, Rapoport A (2001). Interrelations of the yeast *Candida utilis* and Cr(VI): metal reduction and its distribution in the cell and medium. *Process Biochemistry*.

[11] Morales-Barrera L, Guillén-Jiménez FDM, Ortiz-Moreno A (2008). Isolation, identification and characterization of a *Hypocrea tawa* strain with high Cr(VI) reduction potential. *Biochemical Engineering Journal*.

[12] Srivastava S, Thakur IS (2006). Isolation and process parameter optimization of *Aspergillus* sp. for removal of chromium from tannery effluent. *Bioresource Technology*.

[13] Fernández PM, Fariña JI, Figueroa LIC (2010). The significance of inoculum standardization and cell density on the Cr(VI) removal by environmental yeast isolates. *Water, Air, and Soil Pollution*.

[14] Fernández PM, Figueroa LIC, Fariña JI (2010). Critical influence of culture medium and Cr(III) quantification protocols on the interpretation of Cr(VI) bioremediation by environmental fungal isolates. *Water, Air, and Soil Pollution*.

[15] Cheung KH, Gu JD (2003). Reduction of chromate (CrO_4_ 
^2−^) by an enrichment consortium and an isolate of marine sulfate-reducing bacteria. *Chemosphere*.

[16] Acevedo-Aguilar FJ, Espino-Saldaña AE, Leon-Rodriguez IL (2006). Hexavalent chromium removal in vitro and from industrial wastes, using chromate-resistant strains of filamentous fungi indigenous to contaminated wastes. *Canadian Journal of Microbiology*.

[17] QuiIntana M, Curutchet G, Donati E (2001). Factors affecting chromium(VI) reduction by *Thiobacillus ferrooxidans*. *Biochemical Engineering Journal*.

[18] Mclean J, Beveridge TJ (2001). Chromate reduction by a *Pseudomonad* isolated from a site contaminated with chromated copper arsenate. *Applied and Environmental Microbiology*.

[19] Megharaj M, Avudainayagam S, Naidu R (2003). Toxicity of hexavalent chromium and its reduction by bacteria isolated from soil contaminated with tannery waste. *Current Microbiology*.

[20] Pal A, Paul AK (2004). Aerobic chromate reduction by chromium-resistant bacteria isolated from serpentine soil. *Microbiological Research*.

[21] Bai R S, Abraham TE (2001). Biosorption of Cr (VI) from aqueous solution by *Rhizopus nigricans*. *Bioresource Technology*.

[22] Sağ Y, Aktay Y (2002). Kinetic studies on sorption of Cr(VI) and Cu(II) ions by chitin, chitosan and *Rhizopus arrhizus*. *Biochemical Engineering Journal*.

[23] Tewari N, Vasudevan P, Guha BK (2005). Study on biosorption of Cr(VI) by *Mucor hiemalis*. *Biochemical Engineering Journal*.

[24] Farrell SO, Ranallo RT (2000). *Experiments in Biochemistry. A Hands-On Approach*.

[25] Wittbrodt PR, Palmer CD (1996). Effect of temperature, ionic strength, background electrolytes, and Fe(III) on the reduction of hexavalent chromium by soil humic substances. *Environmental Science and Technology*.

[26] Wang YT, Xiao C (1995). Factors affecting hexavalent chromium reduction in pure cultures of bacteria. *Water Research*.

[27] McLean JS, Beveridge TJ, Phipps D (2000). Isolation and characterization of a chromium-reducing bacterium from a chromated copper arsenate-contaminated site. *Environmental Microbiology*.

[28] Das S, Chandra AL (1990). Chromate reduction in *streptomyces*. *Experientia*.

[29] Pal A, Dutta S, Mukherjee PK, Paul AK (2005). Occurrence of heavy metal-resistance in microflora from serpentine soil of Andaman. *Journal of Basic Microbiology*.

[30] Desai C, Jain K, Madamwar D (2008). Evaluation of *in vitro* Cr(VI) reduction potential in cytosolic extracts of three indigenous *Bacillus* sp. isolated from Cr(VI) polluted industrial landfill. *Bioresource Technology*.

[31] Kwak YH, Lee DS, Kim HB (2003). *Vibrio harveyi* nitroreductase is also a chromate reductase. *Applied and Environmental Microbiology*.

[32] Mazoch J, Tesařík R, Sedláček V, Kučera I, Turánek J (2004). Isolation and biochemical characterization of two soluble iron(III) reductases from *Paracoccus denitrificans*. *European Journal of Biochemistry*.

